# GRPR-targeted Protein Contrast Agents for Molecular Imaging of Receptor Expression in Cancers by MRI

**DOI:** 10.1038/srep16214

**Published:** 2015-11-18

**Authors:** Fan Pu, Jingjuan Qiao, Shenghui Xue, Hua Yang, Anvi Patel, Lixia Wei, Khan Hekmatyar, Mani Salarian, Hans E. Grossniklaus, Zhi-Ren Liu, Jenny J. Yang

**Affiliations:** 1 Departments of Chemistry and Biology, Center for Diagnostics & Therapeutics, Georgia State University, Atlanta, GA 30303; 2 Department of Ophthalmology, Emory University, Atlanta, GA, 30322; 3 Bio-imaging Research Center, University of Georgia, Athens, GA, 30602.

## Abstract

Gastrin-releasing peptide receptor (GRPR) is differentially expressed on the surfaces of various diseased cells, including prostate and lung cancer. However, monitoring temporal and spatial expression of GRPR *in vivo* by clinical MRI is severely hampered by the lack of contrast agents with high relaxivity, targeting capability and tumor penetration. Here, we report the development of a GRPR-targeted MRI contrast agent by grafting the GRPR targeting moiety into a scaffold protein with a designed Gd^3+^ binding site (ProCA1.GRPR). In addition to its strong binding affinity for GRPR (K_d_ = 2.7 nM), ProCA1.GRPR has high relaxivity (r_1_ = 42.0 mM^−1^s^−1^ at 1.5 T and 25 °C) and strong Gd^3+^ selectivity over physiological metal ions. ProCA1.GRPR enables *in vivo* detection of GRPR expression and spatial distribution in both PC3 and H441 tumors in mice using MRI. ProCA1.GRPR is expected to have important preclinical and clinical implications for the early detection of cancer and for monitoring treatment effects.

Prostate cancer is the leading cause of tumor-related death in men in the Western world. According to the estimation from the National Cancer Institute, about 240,890 new cases occurred in 2011, with 33,720 associated deaths. One of the major reasons for the high mortality is the limitations of current methods for the early detection, accurate diagnosis, and treatment monitoring capabilities of this disease. Current determination of the histological type of prostate cancer by invasive clinical diagnostic procedures, e.g., biopsy, may only be achieved several years after the initial finding of elevated levels of prostate-specific antigen (PSA) due to limitations of existing imaging methodologies for non-invasive assessment of the tumor. Similarly, lung cancer lacks an accurate and non-invasive detection method, despite being the leading cause of cancer related death in men and women in the United States, with 86,740 deaths in men and 70,759 in women in 2012. Thus, there is an urgent need to develop sensitive and accurate non-invasive imaging methods to assess cancer states *in vivo* using biomarkers, and subsequently monitor tumor progression, metastasis, and treatment effectiveness with high specificity.

Biomarkers, such as gastrin-releasing peptide (GRP) receptor (GRPR), were suggested to be attractive early cancer indicators^1^,^2^. GRPR was reported to be overexpressed on the surfaces of various human cancers, including breast, colon, lung, and prostate cancer^3^,^4^,^5^. Elevated GRPR expression was found on the cell membrane of prostatic intraepithelial neoplasia, primary prostate cancer, invasive prostatic carcinoma, and androgen-independent human cancer cells as well as well-differentiated and metastatic prostate cancers^4^. In contrast, GPPR has very limited expression in normal prostate^4^. High levels of GRPR were observed in prostate and breast tissues during malignant transformation^3^,^4^.

GRPR is a member of the mammalian bombesin receptor family with the capacity to bind short peptides, such as GRP or bombesin^5^. *In vivo* imaging of tumor cells and tumor-bearing mice with GRPR expression have been reported by conjugating residues 7–14 from the C-terminal of GRP and bombesin to NIR dyes^6^,^7^,^8^, quantum dots^9^, and SPECT or PET probes^10^,^11^,^12^,^13^. Preclinical and clinical uses of bombesin-based radiopharmaceuticals have also been reported^14^,^15^,^16^,^17^. These studies justify the potential applications of GRPR-targeted imaging and therapeutic reagents based on bombesin binding. However, monitoring differential expression of GRPR in different types of cancers using non-invasive and non-radioactive MR molecular imaging has not yet to be achieved.

As one of the leading diagnostic techniques in clinical and preclinical settings, MRI has the advantage of capturing three dimensional anatomical images with increased body depth without ionized radiation. Moreover, it enables the non-invasive and repetitive assessment of biological processes in the same living subject at different time points, significantly reducing the number of animals required and the subsequent cost associated with preclinical studies. Clinical TNM (Tumor, Node, and Metastasis) staging is commonly used in the USA. MRI is more accurate than CT, ultrasound and digital rectal examination in the assessment of unilateral or bilateral diseases (stage T2), extracapsular extension and invasion of seminal vesicles (stage T3), as well as invasion of adjacent structures (stage T4)^18^. However, it remains as a challenge for MRI to follow the recurrence and metastasis of prostate cancer upon PSA levels increasing after drug treatment.

Molecular imaging of cancer biomarkers using MRI potentially improves our understanding of disease states and effects of drug treatment^19^. However, one of the major hurdles for the application of MRI to assess specific disease markers for diagnosis and monitoring drug effects is the lack of contrast agents capable of enhancing the contrast between normal tissues and tumors with high relaxivity, targeting capability, tumor penetration and reduced toxicity. Clinical Gd^3+^-containing MRI contrast agents, such as Gd-DTPA, have relaxivities below 5 mM^−1^s^−1^. Additions of targeting moieties to this class of contrast agents fail to provide information about changes in biomarkers due to low relaxivity and low biomarker expression at nM or lower. In addition, high dosages (0.1–0.2 mmol/kg) are required to provide sufficient *in vivo* imaging contrast. Additional concerns about the risk of nephrogenic systemic fibrosis associated with metal toxicity must be addressed^20^. Taken together, there is an urgent need to develop MRI contrast agents with significantly improved relaxivities and targeting capabilities to monitor changes of disease biomarkers with sufficient temporal and spatial resolution.

We have developed protein-based MRI contrast agents (ProCAs) by introducing a Gd^3+^ binding site into a scaffold protein, CD2 (ProCA1), with high relaxivities compared to clinically available MRI contrast agents both *in vitro* and *in vivo*^21^,^22^,^23^,^24^. Although using short peptide ligands as targeting moieties has advantages for tumor penetration compared to larger antibodies, peptides often have less specificity as well as *in vivo* instability due to the lack of defined structures. In the present study, we report the development of GRPR-targeted reagent with improved targeting capability by a grafting approach. We then demonstrate that the designed GRPR-targeting reagent (ProCA1.GRPR) has the unique capacity to selectively enhance the MRI signal of xenografted prostate tumor depending on the expression levels of GRPR *in vivo* in mice due to its improved relaxivity, targeting capability and specificity. We also show that ProCA1.GRPR has no detectable acute toxicity.

## Results

### Design of Protein-based MRI contrast agent with improved targeting capability

**Figure 1:**
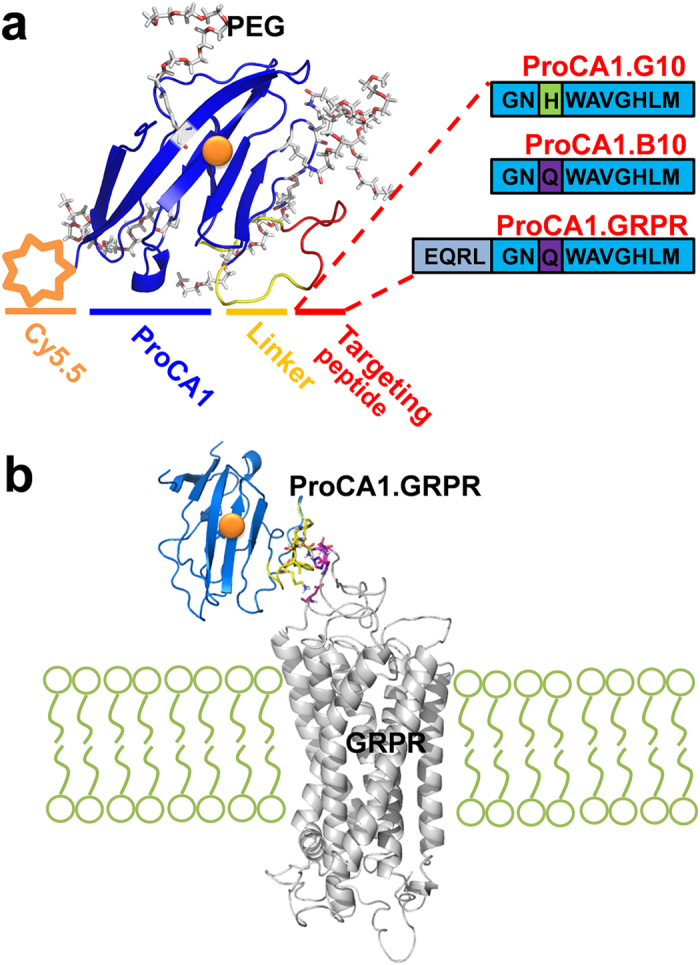
Modeled structures of GRPR-targeted ProCAs and *in silico* docking of GRPR-targeted ProCAs to GRPR. (**a**) Optimizing GRPR targeting peptide for molecular imaging by MRI. The modeled structure of ProCA1 variants (blue) with grafted GRPR targeting peptide from GRP or bombesin (red). ProCA1 is a protein-based MRI contrast agent derived from domain 1 of rat CD2 with several mutations to form a gadolinium (orange) binding pocket on its surface. ProCA1.B10 and ProCA1.G10 contain 10 amino acids at the C-terminal of bombesin and GRP, respectively. They share the same peptide sequence except one residue (H/Q) difference which is related to the GRP/bombesin-GRPR binding affinity. ProCA1.GRPR contains the whole sequence of bombesin with 14 amino acids. The ProCA1 and GRPR targeting peptide were connected through a flexible linker (yellow). These GRPR-targeted contrast agents are also PEGylated and conjugated with Cy5.5. (**b**) *In silico* docking of GRPR-targeted ProCAs to GRPR by HADDOCK.

Figure 1 shows the modeled structure of ProCA1 with three designed GRPR targeting moieties (ProCA1.G10, ProCA1.B10 and ProCA1.GRPR also named as ProCA1.B14) flanked by two glycine linkers grafted at position 52 of the host protein. These GRPR-targeted protein contrast agents were designed with the following considerations. First, ProCA1 is a protein-based MRI contrast agent with a designed gadolinium binding site in the scaffold protein, domain 1 of rat CD2. We have previously shown that the relaxivity of ProCA1 is significantly greater than that of clinically approved contrast agent, Gd-DTPA^21^. Second, our previous studies on applying a grafting approach for ligand recognition suggested that peptide ligands grafted into a scaffold protein are able to retain native conformations^25^,^26^,^27^. We reasoned that the GRP peptide grafted into a scaffold protein will have an improved capacity for molecular recognition compared to short peptide fragments lacking a defined conformation in solution, and will also reduce risk of degradation^26^. Third, three targeting moieties were designed to test the contribution of ligand length and H to Q mutation in bombesin on binding affinity. We hypothesize that grafting a full length sequence (14 residues) of bombesin (named ProCA1.GRPR) rather than 10 residue fragment from the C-terminal of GRP or Bombesin (named ProCA1.G10 or ProCA1.B10, respectively) in ProCA1 would bestow greater GRPR binding affinity. Fourth, we further modify the surface of ProCA1.GRPR by PEGylation to increase protein solubility, stability, and *in vivo* retention time. Fifth, a near-infrared (NIR) dye was conjugated to ProCA1 variants to provide NIR fluorescence modality to verify molecular imaging by MRI (Fig. 1a).

The addition of a targeting moiety does not alter conformation and metal binding capacity. Three designed targeting reagents were bacterially expressed and purified. Far UV CD spectra suggested that the secondary structures of ProCA1 variants are similar to that of ProCA1 and are not altered by either the presence or absence of Gd^3+^ (Supplementary Fig. 1). The tryptophan fluorescence emission maxima of ProCA1 variants with addition of the targeting sequences are also near 330 nm, which is similar to ProCA1 but largely blue shifted compared to free tryptophan excited at 280 nm. This result suggests that the aromatic tryptophan residues remain well buried in the native scaffold protein (Supplementary Fig. 1).

The Gd^3+^ binding capabilities of GRPR-targeted variants were determined by competition with Fluo-5N^21^. The fluorescence emission signals of Fluo-5N gradually increased upon the addition of Gd^3+^ in the solution. The calculated K_d_ value of Fluo-5N to Gd^3+^ is 5.2 × 10^−12^ M (Supplementary Fig. 2). Supplementary Fig. 3a shows that the addition of targeting contrast agent ProCA1.GRPR results in a decrease of the fluorescence emission of Fluo-5N due to competition. All three GRPR-targeted contrast agents exhibit similar Gd^3+^ binding affinity near 10^−12^ M (Table 1).

Zn^2+^ is a major physiological metal ion that participates in the de-chelation of Gd^3+^ from clinical MRI contrast agents *in vivo*^20^. Therefore, it is crucial to evaluate the metal selectivity for Gd^3+^ over Zn^2+^ in developed MRI contrast agents. Table 1 summarizes the Zn^2+^ affinities to ProCA1 variants that were determined by the competition between ProCA1 variants and Fluozin-1 (Supplementary Fig. 3b). All of three GRPR-targeted contrast agents exhibit Zn^2+^ binding affinity around 10^−6^ M. Importantly, the Gd^3+^ selectivities over Zn^2+^ (log (K_Gd_/K_Zn_)) for the three targeted contrast agents are significantly greater than that of the clinically approved contrast agent, DTPA of 4.2. The Gd^3+^ selectivity over Zn^2+^ for ProCA1, ProCA1.GRPR, ProCA1.B10 and ProCA1.G10 is 5.4, 6.3, 6.3 and 6.5, respectively (Table 1).

### Relaxivity measurement of ProCA1 variants

**Figure 2:**
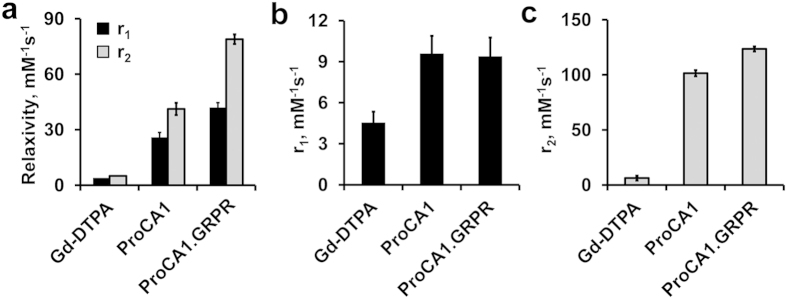
The relaxivities of ProCA1 variants. (**a**) The relaxivities (r_1_ and r_2_) of Gd-DTPA, ProCA1 and ProCA1.GRPR at 1.4 T and 25 °C. (**b**) r_1_ of Gd-DTPA, ProCA1 and ProCA1.GRPR at 7 T and 37 °C. (**c**) r_2_ of Gd-DTPA, ProCA1 and ProCA1.GRPR at 7 T and 37 °C. Data are expressed as mean ± s.d.

The r_1_ and r_2_ relaxivity values of ProCA1 variants were measured at 25 °C at 1.4 T (Fig. 2a). As shown in Table 1, all GRPR-targeted ProCA1 variants have r_1_ relaxivity values between 42.0–49.2 mM^−1^s^1^. These values are at least 12 fold greater than those of Gd-DTPA (3.5 mM^−1^s^−1^). Addition of full length bombesin to ProCA1 (named ProCA1.GRPR) led to an increase of r_1_ relaxivity from 25.9 to 42.0 mM^−1^s^1^. In addition, ProCA1.GRPR has r_1_ of 9.4 ± 1.4 mM^−1^s^−1^ and r_2_ of 123.5 ± 2.3 mM^−1^s^−1^ at 37 °C and 7 T (Fig. 2b,c).

### Determination of the targeting and binding affinities of ProCA1 variants to GRPR on cancer cells

**Figure 3:**
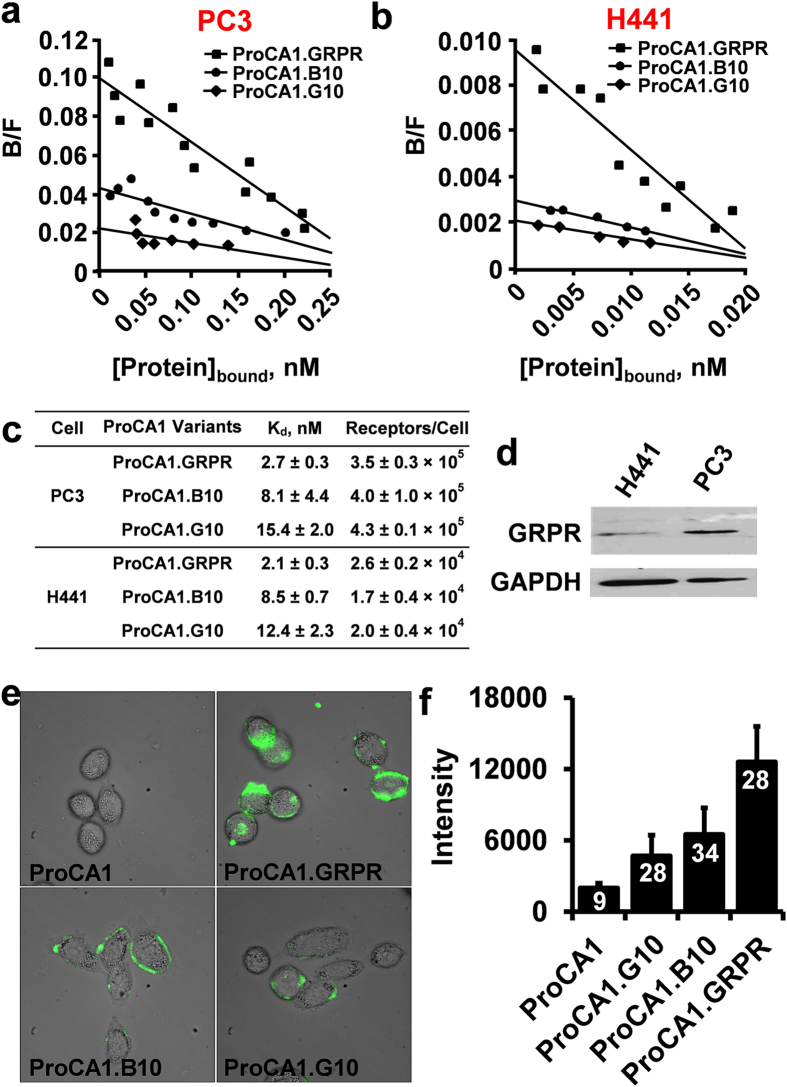
Characterization of the interaction between ProCA1.GRPR and GRPR on tumor cells. (**a, b**) Determination of the binding affinity of ProCA1 variants to GRPR in PC3 (**a**) and H441 cells (**b**) by Scatchard plot. (**c**) Summary of GRPR binding affinity of ProCA1 variants in PC3 and H441 cells. ProCA1.GRPR exhibits the highest GRPR binding affinity among the three designed protein MRI contrast agents in both PC3 and H441 cells. The GRPR numbers (B_max_) per PC3 cell were 13–23 times higher than those of H441. (**d**) Western blot shows that GRPR has a higher level of expression in PC3 cells than that of H441 cells. The images a,b,d are representatives of three independent experiments. (e) Fluorescence imaging of PC3 cells incubated with fluorescein-labeled ProCA1 variants (green). (**f**) Fluorescence intensity of PC3 cells incubated with different ProCA1 variants. The mean and standard derivation (error bar) of fluorescence intensity were quantified from 9, 28, 34 and 28 cells after incubation with ProCA1, ProCA1.G10, ProCA1.B10, and ProCA1.GRPR, respectively. Data are expressed as mean ± s.d.

We chose two independent cell lines, PC3 and H441, to evaluate the targeting properties of gastrin-releasing peptide receptor (GRPR)^28^,^29^. PC3 is an androgen independent human prostate cancer cell line and H441 is a human lung cancer cell line. We first quantitatively determined the expression levels of GRPR on both cancer cell lines using ELISA coupled with the use of the Scatchard Plot (equation 3 and Fig. 3a–c). The GRPR expression levels ([B_max_]) are approximately 4 × 10^5^ and 2 × 10^4^ receptors/cell for PC3 and H441, respectively. Consistent with these results, data from Western blot also revealed that GRPR expression is significantly greater for PC3 cells than that for H441 cells (Fig. 3d). As shown in Fig. 3c, ProCA1.GRPR has the highest GRPR binding affinity with a dissociation constant (K_d_) of 2.7 nM. GRPR binding affinity for ProCA1.B10 and ProCA1.G10 are 3 and 5.7 fold lower than that of ProCA1.GRPR, respectively. Consistent with ELISA, ProCA1.GRPR shows the highest fluorescence staining in PC3 cells, compared with ProCA1, ProCA1.B10 and ProCA1.G10 (Fig. 3e,f).

### Molecular imaging of ProCA1.GRPR targeting GRPR in tumor-bearing mice

We chose ProCA1.GRPR for its high GRPR binding affinity as an agent to image GRPR in xenograft tumors in mice. ProCA1 and ProCA1.GRPR were PEGylated to increase the solubility of the protein and decrease its immunogenicity. An MRI/NIR dual modality imaging reagent was created by conjugating ProCA1.GRPR with NIR dye Cy5.5. The metal binding affinity and relaxivity of modified ProCA1.GRPR remained unchanged.

**Figure 4:**
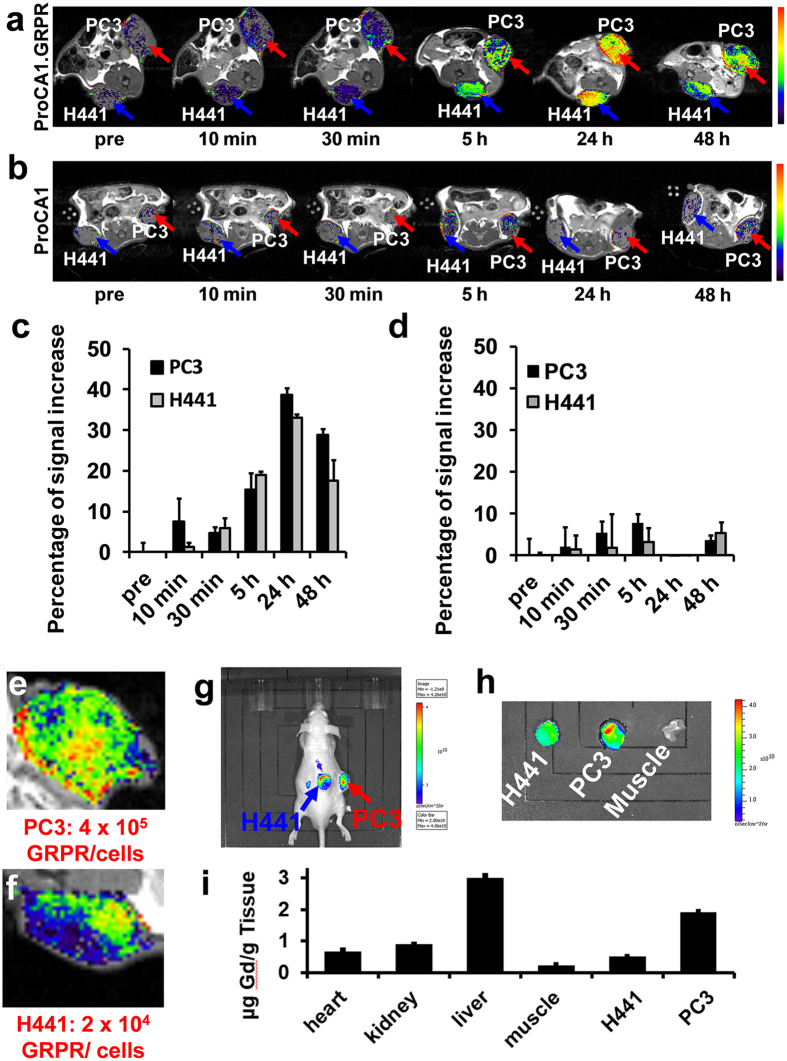
Molecular imaging of the GRPR biomarker on mice xenografted with PC3 and H441 cells by T1-weighted spin echo MR imaging and NIR imaging. (**a**) T1-weighted spin echo MR imaging of ProCA1.GRPR targeting GRPR in PC3 (red arrow) and H441 (blue arrow) xenografted mice tumors. (**b**) T1-weighted spin echo MR imaging of non-targeted ProCA1 in PC3 (red arrow) and H441 (blue arrow) xenografted mice tumors. (**c**) Percentage of increase in MRI signal in PC3 and H441 tumor before and after injection of ProCA1.GRPR. PC3 tumor exhibits a 1.5 fold higher MRI enhancement compared with H441 tumor at 48 hours post injection of ProCA1.GRPR. (**d**) Percentage of MRI signal increase in PC3 and H441 tumor before and after injection of non-targeted ProCA1. As a negative control, non-targeted ProCA1 does not have significant enhancement in both tumors after injection of non-targeted ProCA1. Images a-d are representatives of three independent experiments. Data are expressed as mean ± s.d. of percentage increase of tumor intensity values measured from different MRI slides of H441 or PC3 tumor in each representative animal from three independent experiments. (**e**, **f**) MRI shows heterogeneous enhancement in PC3 (**e**) and H441 tumors (**f**) after injection of ProCA1.GRPR. (**g**) NIR imaging of PC3 and H441 xenografted mice tumors 48 hours post ProCA1.GRPR injection. (**h**) NIR imaging of isolated PC3, H441 tumors and muscle 48 h post injection of ProCA1.GRPR. PC3 tumor shows higher NIR intensity compared to H441 tumor. (**i**) Gd^3+^ distribution in different tissues 48 hours post ProCA1.GRPR injection. Data are expressed as mean ± s.d.

**Figure 5:**
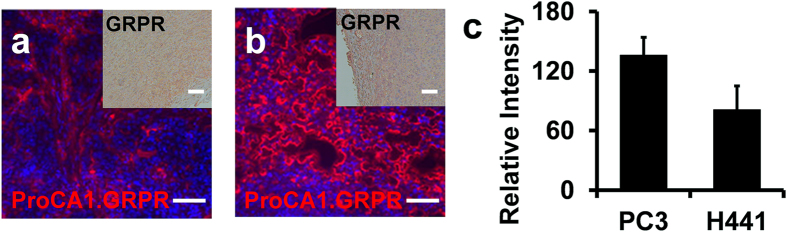
ProCA1.GRPR distribution and GRPR expression in xenografted tumor tissue. (**a,b**) Immunofluorescence staining of ProCA1.GRPR targeting to GRPR on H441 (**a**) and PC3 tumors (**b**) in xenografted mice. The red color indicates the staining of ProCA1.GRPR while the blue color represents nucleus staining. Insert: Immunohistochemistry staining of GRPR expression on H441 (**a**) and PC3 tumor (**b**) from xenografted mice. In comparison with H441 tumors, GRPR shows stronger expression (brown color) on PC3 tumor. (**c**) Fluorescence intensity of ProCA1.GRPR staining in PC3 and H441 tumors. Data are expressed as mean ± s.d. Scale bar = 100 μm. The images a-c are representatives of three independent experiments.

Figure 4 shows the MR and NIR imaging of the mice before and after tail vein administration of ProCA1.GRPR at a dose 8-fold lower than the clinical injection dosage of Gd-DTPA. Both PC3 and H441 tumors were inoculated in the left and right flanks of mice. Consistent with results of the cultured cell studies (Fig. 3), GRPR expression level in PC3 tumor tissue is significantly higher than that in H441 tumor tissue revealed by IHC staining (Fig. 5a,b). Both tumor regions exhibit increases in contrast enhancement from 30 minutes to 48 hours post injection of GRPR-targeted contrast agent, ProCA1.GRPR (Fig. 4a). By comparison, injection of ProCA1 without the GRPR targeting moiety or use of the clinical contrast agent Gd-DTPA to the tumor bearing mice did not result in any significant MRI contrast enhancement in tumor regions under the same conditions (Fig. 4b). PC3 and H441 tumors show 15–20% signal increase at 5 hour post injection of ProCA1.GRPR. The overall MRI enhancement in PC3 tumors is significantly higher than that in H441 tumors at both 24 and 48 hours post injection (approximately 1.5 fold at 48 hours). We also observed NIR fluorescence emission for both PC3 tumor and H441 tumors 48 hours post injection of ProCA1.GRPR (Fig. 4g). *Ex vivo* NIR imaging of tumor tissues revealed that tissues of PC3 tumor indeed have stronger NIR fluorescence than that of H441 tumor (Fig. 4h). This result is consistent with MR imaging studies, which further supports their GRPR targeting capability.

We monitored both Gd^3+^ (Fig. 4i and Supplementary Fig. 4) and protein (Fig. 5) content in tumor-bearing mice after molecular imaging. As shown in Fig. 4i, Gd^3+^ content in PC3 tumor is about three-fold greater than that in H441 tumor. Gd^3+^ contents in both PC3 and H441 tumors are significantly greater than that in muscle tissue that lacks the biomarkers. Intensity of immunofluorescence staining of ProCA1.GRPR in PC3 tumor tissue showed about 2-fold greater than that of H441 tumor tissues (Fig. 5c). The results from both Gd^3+^ content analyses and ProCA1.GRPR staining are in a good agreement with non-invasive MRI contrast enhancement via molecular imaging, demonstrating the strong capability of our developed MRI contrast agent ProCA1.GRPR to monitor GRPR receptor expression levels in tumors in live mice.

### Heterogeneous distribution of GRPR expression in tumor tissue

We further examined the capacity of our developed contrast agent for tumor penetration, which is essential for quantifying spatial distribution of biomarkers. As shown in Supplementary Fig. 5, ProCA1.GRPR is distributed in regions encompassing the entire tumor, and the agent clearly penetrates the tumor vessels as shown by CD31 staining. This result is consistent with our previous studies on the tumor penetrating capability of targeted ProCAs^30^, suggesting that ProCA1.GRPR meets the criteria to quantify spatial distribution of GRPR.

It is interesting to note that both PC3 and H441 tumors show clustered heterogeneous MRI enhancement at 24 and 48 hours after injection of ProCA1.GRPR (Fig. 4e,f). IHC staining verified the heterogeneous distribution of ProCA1.GRPR in PC3 tumors (Supplementary Fig. 6). Thus, our developed MRI contrast agent ProCA1.GRPR enables real time visualization of the differential spatial distribution of GRPR that depends on tumor types and tissue organization. PC3 tumors express GRPR in both the surrounding edge and center of the xenografted tumors whereas H441 tumors have a lower GRPR expression that is mainly expressed in the internal regions.

### Biodistribution and pharmacokinetic studies

Figure 4i shows the gadolinium contents detected by ICP-OES analysis in different organs of tumor-bearing mice post injection of ProCA1.GRPR. In addition to relatively higher distribution in PC3 tumor tissue, ProCA1.GRPR was also largely distributed in liver and kidney.

To determine the pharmacokinetics of ProCA1.GRPR, we obtained plasma samples from the mice at different time points post injection of ProCA1.GRPR or GdCl_3_. Those samples were measured for total Gd^3+^ using an ICP-OES. As shown in Supplementary Fig. 7, ProCA1.GRPR concentration declined rapidly during the distribution phase, (t_1/2α_ = 0.5 hour) and more slowly during the elimination phase, (t_1/2β_ = 8.6 hours). The mean total clearance was 6.6 ± 1.0 ml/h/kg. The steady state volume distribution (V_dss_ = 0.06 l/kg) indicates that ProCA1.GRPR was distributed in the extracellular extravascular space.

### Toxicity study of ProCA1.GRPR in mice

To probe the clinical acute toxicity of ProCA1.GRPR in mice, we injected PEGylated ProCA1.GRPR in normal mice and dissected the mice 2 days post injection. The blood samples were then collected to assess toxicity by clinical chemistry. In comparison with mice injected with saline, ProCA1.GRPR did not show significant difference in acute toxicity (Supplementary Table 1). We performed histological analysis of different organs by H&E staining. Comparing the pathology of control mice and the mice injected with ProCA1.GRPR, we concluded that there is no significant abnormal morphology in different organs (Supplementary Fig. 8).

## Discussion

Exciting progress in the molecular imaging of disease biomarkers for tumor formation and biomarker expression has been achieved using several imaging methods such as NIR fluorescence and PET. While these imaging modalities are highly sensitive, their applications are often limited by spatial imaging resolution along with tumor penetration due to the large size of antibodies (~150 kDa). We and others have shown that antibodies tend to be inadequately distributed, clustering near tumor blood vessels even 24 hours-post injection in mice^30^. On the other hand, peptides derived from natural ligands used to target biomarkers, such as GRP often exhibit reduced specificity/affinity as well as *in vivo* biostability due to lacking a defined structure^26^. Therefore, there is an urgent need for the development of a non-invasive imaging agent capable of quantitatively determining expression level and spatial distribution of biomarkers^31^.

MRI is the most desirable imaging modality for the molecular imaging of biomarkers due to its capacity for three-dimensional imaging of soft tissues with high resolution, without depth limitation or use of ionized radiation. While MRI scanners are popular worldwide with rapid instrumental progress, MRI is heavily underutilized for both clinical and preclinical application. That is mainly due to the limitations of approved contrast agents with low relaxivity (approximately 3.5 mM^−1^s^−1^), which do not permit molecular imaging for monitoring biomarkers such as receptors with concentrations usually at the μM or nM range. Tremendous effort has been devoted to develop MRI contrast agents with improved relaxivity and targeting capability to improve sensitivity and specificity for disease biomarkers^32^,^33^,^34^,^35^,^36^,^37^,^38^,^39^,^40^. Despite great success in tumor detection utilizing fluorescence and PET, molecular imaging in clinical MRI is now still in its infancy.

To address the urgent need for early and accurate diagnosis of prostate cancer, we aimed to develop a protein MRI contrast agent capable of monitoring the expression of GRPR. GRPR is an important biomarker for many types of diseases, such as prostate cancer, cervical cancer, lung cancer, uveal melanoma and pruritus^41^,^42^. Short peptides like GRP, bombesin and their analogues have been linked to radioactive isotopes as a means to label GRPR in prostate cancer and potentially to treat prostate cancer^10^,^11^,^12^,^13^,^43^,^44^,^45^,^46^,^47^,^48^. However, the molecular imaging of GRPR using MRI is limited substantially due to the lack of contrast agents with desired sensitivity due to relaxivity and specificity related to biomarker targeting capability.

We previously reported our studies in developing protein-based MRI contrast agents with targeting capability for molecular imaging of GRPR by engineering a 10-residue gastrin releasing peptide introduced at different regions of ProCA1 (ProCA1.GRP)^26^. We have shown that contrast agents with a targeting peptide grafted in the loop region of ProCA1 have qualitatively better *in vitro* targeting abilities than those in which it is fused to the C-terminal of ProCA1 for GRPR expressed by both PC3 and DU145 tumor cells^26^. While ProCA1.GRP has some MRI enhancement at tumor regions after intratumoral injection, it failed to show any significant tumor enhancement in mice xenografted with PC3 cells by tail vein injection of ProCA1.GRP (Supplementary Fig. 9).

In this paper, we have achieved the development of GRPR targeting contrast agents based on ProCA1 using our developed grafting approach. The per Gd^3+^ relaxivity for ProCA1.GRPR is 42.0 ± 2.6 at 1.4 T and 25 °C, more than 10 times higher r_1_ than that of clinically utilized MRI contrast agents, such as DTPA (around 3.5 mM^−1^s^−1^). Additionally, ProCA1 and ProCA1.GRPR have high r_1_ and r_2_ at 37 °C and 7 T. Interestingly, the addition of targeting peptides to ProCA1 significantly enhanced r_1_ and r_2_ at 1.4 T. The dramatic increase of relaxivities could result from several possible factors. First, the addition of targeting peptide may place the metal binding site closer to the barycenter of the scaffold proteins and also enhance second shell water contribution. Fulton *et al.* have reported that enhancement of the relaxivities of Gd-based MRI contrast agents can be achieved by placing the metal ion at the barycentre of the dendritic gadolinium complexes to improve coupling between the Gd-water vector and the tumbling motion of the complex as demonstrated^49^,^50^. Second, water exchange rate and secondary and outer sphere water contribution increased upon grafting targeting peptide in ProCA1 could also increase the relaxivity. Third, increase in water number upon addition of targeting moiety could cause r_1_ and r_2_ increase. This is less likely based on the fact that metal binding affinity is not dramatically changed.

To develop MRI contrast agents for *in vivo* application, contrast agents with strong Gd^3+^ stability are highly desirable. The stability of the contrast agents are characterized by thermodynamic stability, conditional stability and kinetic stability. We have shown here that the Zn^2+^ selectivity of ProCA1.GRPR is 2 orders of magnitude higher than that of clinical MRI contest agents (Table 1) that is very encouraging. Consistently, our primary results indicated that in the presence 50 μM of Zn^2+^ or 50 and 500 µM Ca^2+^ does not alter the relaxation rate of ProCA1.GRPR (Supplementary Fig. 3c). On the other hand, ProCA1.GRPR exhibits 4 orders lower thermodynamic stability than that of Omniscan. The thermodynamic stability of clinical contrast agents are ranged from 10^16.85^–10^25.6^ 51. Since the *in vivo* release of Gd^3+^ was reported to be related to nephrogenic systemic fibrosis^20^, we will further optimize the *in vivo* stability, thermodynamic stability and kinetic stability of ProCA1.GRPR to moving to our goal for clinical applications.

Our understanding of the molecular recognition of these ligand peptides by the receptor is largely hampered by the lack of three dimensional structures and challenges associated with membrane proteins^52^. To systematically dissect the key determinants for biomarker targeting, we have quantitatively determined the GRPR binding affinities of the developed contrast agents using Scatchard plot. Among three contrast agents we created, ProCA1.GRPR, with a 14-residue bombesin targeting moiety, shows the highest GRPR binding affinity (K_d_ of 2.7 ± 0.3 nM). This high affinity likely originates from two major factors. First, the Glu residue in the middle of the 10 residues at the C-terminal is likely to play an important role in GRPR affinity. Our binding study for the 10-residue targeting moieties of bombesin (B10) and GRP (G10) revealed that B10 has a K_d_ of 8.1 ± 4.4 nM for GRPR, which is stronger than the K_d_ of 15.4 ± 2.0 nM for G10. Second, comparison of the binding affinities of ProCA1.GRPR and ProCA1.B10 for GRPR revealed that the additional 4 residues at the N-terminal of bombesin increase GRPR affinity about 3 fold. Interestingly, ProCA1.GRPR also has the lowest binding energy for its binding to GRPR by HADDOCK and a modelled structure of GRPR (Fig. 1b). The strongest affinity for the full length bombesin in ProCA1.GRPR is thus likely due to the combination of its ability to maintain a native conformation, additional interaction surface, and contribution of the H to Q mutation optimized for molecular recognition. Previous binding studies using peptide models largely based on radiolabelled peptide and a competition assay that depends on temperature, salt conditions and cell types were somewhat controversial. The 10 amino acids in the C-terminal of bombesin and GRP were reported to be essential for their binding affinity for GRPR^5^ and a full length bombesin peptide was reported to have a stronger GRPR binding affinity than that of 9 amino acid fragment from the C-terminal of bombesin/GRP^53^,^54^. A full length bombesin peptide was also reported to have a stronger inhibition capability than that of a 10 amino acid fragment from the C-termini of bombesin/GRP in their binding to GRPR transiently expressed in BALB 3T3 cells^53^,^54^. Receptor binding capabilities of GRPR targeting peptides conjugated to imaging moieties were shown to be also related to the linkers^6^,^55^,^56^.

ProCA1.GRPR exhibits several unique capabilities for *in vivo* molecular imaging of GRPR for cancer detection. First, ProCA1.GRPR enabled unprecedented sensitivity for the non-invasive detection of GRPR at low expression levels (2 × 10^4^ receptors/cell) in H441 tumors and middling expression (4 × 10^5^ receptors/cell) in PC3 prostate tumor using MRI for tumor xenografted mice with 8-fold lower injection dosage than that of Gd-DTPA. Second, MRI enhancements for both types of tumors by ProCA1.GRPR are correlated with their receptor expression level as supported by detailed metal and histological analysis as well as *ex vivo* NIR imaging. Third, although enhanced permeability and retention (EPR) effects allows macromolecules with a size larger than 40 kDa to accumulate in tumor^57^, the inadequate tumor penetration of these large molecules limited their application in molecular imaging. Dreher *et al.*,^58^ demonstrated that fluorescein-labeled dextran with a molecular weight of 40–70 kDa was able to accumulate in tumor within 30 min; however, these dextran mainly accumulated along the blood vessels. Conversely, dextran with a molecular weight between 4.7–10 kDa can penetrate deeply into the tumor within 30 min^58^, which increases their potential for tumor imaging and treatment. Due to the unique advantage of ProCAs for molecular imaging with their small size (2–3 nm), ProCAs were shown to have much higher tumor penetration compared with antibodies^30^. Our results strongly demonstrate that ProCA1.GRPR is capable of semi-quantitatively evaluating GRPR expression levels between different tumor cells. Additionally, since the pixel resolution of MRI can easily achieve a sub-millimeter level^59^, ProCA1.GRPR could provide great spatial resolution to evaluate GRPR expression levels in tumor non-invasively.

Limited observations were reported for the heterogeneous distribution of other biomarkers such as integrin α_v_β_3_^60^,^61^, VEGF/VEGFRs^62^, and protease activity^63^ using ultra small superparamagnetic iron oxide particles^60^, paramagnetic nanoparticles^61^, ^124^I-labeled anti-VEGF monoclonal antibody^62^, and fluorescence probe^63^. An MRI para-CEST agent was reported for mapping tumor pH inoculated in mice. The pH map obtained by this agent shows a great heterogeneity with good spatial and temporal resolution^64^. Angiogenesis-targeted iron oxide-based nanoparticles were used to report the heterogeneous distribution of α_v_β_3_ integrin largely confined at blood vessel for angiogenesis based on T_2_ effect^60^. Our developed GRPR-targeted MRI contrast agents enable us for the first time to observe differential and heterogeneous distribution of GRPR expression levels in prostate tumor PC3 and lung tumor H441 models. Unlike these reported studies, our contrast agent enables the targeting of GRPR outside of tumor vessels with a brighter effect and improved *in vivo* pharmacokinetics. This study opens a new avenue for future development and applications for probing the temporal and spatial changes of various biomarkers by MRI.

## Conclusions

We have developed a GRPR-targeted MRI contrast agent by grafting a full length bombesin sequence into a scaffold protein with a designed Gd^3+^ binding site. Of three of GRPR-targeted ProCAs, ProCA1.GRPR displays the strongest binding affinity with K_d_ of 2.7 nM. It also has both high relaxivity of r_1_ and r_2_ that is 12–15 folds higher than that of Gd-DTPA. The high relaxivity, improved *in vivo* biodistribubtion and pharmacokinetics enable *in vivo* imaging of GRPR in tumor bearing mice with a significantly reduced injection dose. The MRI enhancement in both PC3 and H441 tumors were correlated with receptor expression levels and spatial heterogeneous expression and were further confirmed by ICP-OES and NIR imaging and histological analysis. The significantly superior metal selectivity of ProCA1.GRPR for Gd^3+^ over Zn^2+^ compared to DTPA, reduced injection dose, and lack of acute toxicity as assessed by blood chemistries and tissue analysis of ProCA1.GRPR suggests the potential for its *in vivo* application. These results suggest the possibility of using MRI to quantitatively trace the dynamic changes of biomarkers during disease development and the possibility in evaluating the progression of prostate cancer and drug treatment effects in the clinical applications.

## Materials and Methods

### Design and production of GRPR-targeted MRI contrast agents

ProCA1 is a protein contrast agent created by engineering a gadolinium binding site into the scaffold protein of domain 1 of rat CD2^21^. GRPR targeting peptides, including full length bombesin variant (ProCA1.GRPR, also called ProCA1.B14), 10 amino acid peptide of C-terminal bombesin (ProCA1.B10) and GRP (ProCA1.G10) were inserted with glycine linkers flanking both ends at position 52 of ProCA1 (Fig. 1) using polymerase chain reaction (PCR). The amplified linear DNA sequences were phosphorylated by PNK (New England Biolabs), ligated by T4-ligase (New England Biolabs) and then used to transfect *E. coli* competent cells DH5α. The single colony grown on the plate was collected and amplified in LB medium with 0.1% ampicillin. The extracted plasmids were sequenced. ProCA1 variants were expressed by *E. coli* BL21DE3 at 25 °C overnight. *E. coli* cell pellets with expressed ProCA1 variants were purified by unfolding with urea. The refolded protein after dialysis was further purified by FPLC equipped with HiTrap Q HP column. The purified protein was then verified by mass spectrometry with expected molecular weight.

### Determination of Gd^3+^ binding affinity of ProCA1 variants

The Ca^2+^ dye Fluo-5N binds Gd^3+^ and produce a fluorescence signal. The binding affinity of ProCA1 variants to Gd^3+^ was determined by a competition method using Gd^3+^ loaded Fluo-5N (Life Technologies). Fluo-5N emission spectrum was monitored from 500 nm to 600 nm when it was excited at 488 nm. The binding affinity of Fluo-5N to Gd^3+^, K_d1_, was determined by a Gd^3+^ titration in Gd^3+^ buffer system which used 1 mM nitrilotriacetic acid (NTA, Sigma) to control the concentration of free Gd^3+^. To calculate the Gd^3+^ affinity to ProCA1 variant, Fluo-5N was mixed with Gd^3+^ at 1:1 ratio. ProCA1 variants were gradually added into the system to compete Fluo-5N binding with Gd^3+^. An apparent dissociation constant, K_app_, was estimated by fitting the fluorescence emission intensity of Fluo-5N at 520 nm with different ProCA1 variant concentrations as a 1:1 binding model. Gd^3 +^ binding affinities of ProCA1 variants, Kd_2_, were calculated with the equation 1:


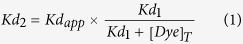


### Determination of Zn^2+^ binding affinity of ProCA1 variants

The Zn^2+^ dye Fluozin-1 (Life Technologies) binds Zn^2+^ and produces a fluorescence signal. The binding affinity of ProCA1 variants to Zn^2+^ was determined by a competition method using Zn^2+^ loaded Fluozin-1^21^. Fluozin-1 emission spectrum was monitored from 500 to 600 nm when it was excited at 495 nm. The binding affinity of Fluozin-1 to Zn^2+^, Kd_1_, was calculated by direct fluorescence titration. To calculate the Zn^2+^ affinity to ProCA1 variants, Fluozin-1 was mixed with Zn^2+^ at 1:1 ratio. Then ProCA1 variants were gradually added into the system to compete Fluozin-1 with Zn^2+^. An apparent dissociation constant, K_app_, was estimated by fitting the fluorescence emission intensity of Fluozin-1 at 515 nm with different ProCA1 variant concentrations as a 1:1 binding model. Zn^2+^ binding affinities of ProCA1 variants, Kd_2_, were calculated with equation 1.

### Relaxivity measurement of ProCA1 variants

The T_1_ and T_2_ relaxation time of ProCA1 variants and Gd-DTPA were measured at 25 °C by 1.4 T Bruker Minispec and 37 °C by 7 T MRI scanner. r_1_ and r_2_ were calculated by equation 2:


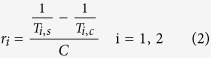


Where T_i,s_ is the T_1_ and T_2_ relaxation time of buffer with contrast agent, T_i,c_ is the T_1_ and T_2_ relaxation time of buffer without contrast agent, and C is the concentration of Gd^3+^.

### Cell imaging of ProCA1 variants targeting GRPR

PC3 cells were incubated with fluorescein-labeled ProCA1 variants at 37 °C for 1 h and then washed with Ringer buffer. All images were taken under the same conditions using Leica imaging system with 63× oil objective lens. The fluorescence intensity of each cell was measured using ImageJ. The final fluorescence intensity of PC3 cells incubated with different ProCA1 variants were plotted as average intensity ± standard derivation.

### Determination of GRPR numbers on cell surface and the ligand binding affinity

The GRPR expression levels of two different cell lines (PC3 and H441) were evaluated by Western blots and ELISA. Indirect ELISA coupled with Scatchard plot was then used to further quantify the GRPR levels in the cell lines and the dissociation constants of the ProCA1 variants to GRPR. Cell lysates of PC3 (from 5 × 10^4^ cells) and H441 (from 5 × 10^4^ cells) were separately precultured in a 96-well plate at 4 °C overnight. After wash with 1× Phosphate Buffered Saline Tween-20 (PBST), the medium was exchanged with 5% BSA to block non-specific binding. ProCA1 variants were added to interact with GRPR in cell lysates. Self-generated rabbit anti ProCA1 antibody was used as the primary antibody. A stabilized goat anti rabbit HRP-conjugated antibody (Pierce) was used as the secondary antibody. The absorbance intensity was detected by the FLUOstar OPTIMA plate reader at an absorbance wavelength of 450 nm. GRPR levels on different cell surface were quantified by Scatchard plot (equation 3).


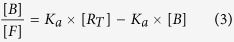


Where K_a_ is the association constant between GRPR and ProCA1 variants, [R_T_] was the concentration of total GRPR on the plate, [B] was the concentration of binding ProCA1variants, [F] was the concentration of free ProCA1 variants.

### MR imaging of ProCA1.GRPR targeting GRPR in mouse tumor

All animal procedures performed in this study were complied in accordance with approved animal protocols by Institutional Animal Care and Use Committee (IACUC) in Georgia State University and University of Georgia. About 5 × 10^6^ PC3 and H441 cells were injected into both flanks of athymic mice to generate the xenograft model. Tumors grew up to 1 cm in diameter near 8 weeks after inoculation. The contrast agents, ProCA1.GRPR or ProCA1 (5 mM, 100 μl), were injected into the mice with grafted tumors by tail vein injection. Mice were scanned on a 7 T MRI scanner using a 38 mm birdcage mouse coil. The mouse was anesthetized with isoflurane during the MR scan process. Spin echo T1-weighted MR images were acquired by spin echo sequences (TR = 400 ms, TE = 14.52 ms) with field of view of 4 × 4 cm, matrix of 128 × 128, signal averaging of 4 and slice thickness of 1 mm.

### NIR imaging of mouse after injection of ProCA1.GRPR

ProCA1 variants were conjugated with NIR dye Cy5.5 (GE Healthcare Life Science) with the ratio 5:1. ProCA1 variants reacted with reduced agent Tris(2-carboxyethyl)phosphine and were then reacted with Cy5.5 in nitrogen saturated solution at dark conditions. Samples were further purified by FPLC^30^. ProCA1.GRPR with conjugated NIR dye exhibited fluorescence excitation and emission maxima at 675 and 694 nm, respectively. The NIR images of mice were acquired using The IVIS Lumina II (Caliper Life Sciences) 48 hours after tail vein injection of MRI contrast agent ProCA1.GRPR. *Ex vivo* NIR images of each organ tissues were also acquired under the same conditions.

### Gd^3+^ distribution in different tissues detected by ICP-OES

After MR and NIR imaging, the mice were dissected and various tissue samples were extracted. One part was encapsulated by optimal cutting temperature compound (O.C.T) and preserved at –80 °C for IHC analysis, while the other was digested by 70% HNO_3_ (Optima) at 110 °C overnight for ICP-OES analysis. The digested solution was evaporated to 1 ml and 2% HNO_3_ (Optima) was added to make the sample volume up to 5 ml. Gd^3+^ concentrations in samples were calculated based on the standard curve measured at 342.246 nm (Supplementary Fig. 4) .

### ProCA1.GRPR target GRPR on PC3 and H441 xenograft tumors by immunofluorescence and immunohistochemistry staining

After MRI and NIR imaging, the mice were sacrificed and various tissues were extracted. The PC3 and H441 tumors were encapsulated by O.C.T and frozen in liquid nitrogen. The frozen specimens were cut in 5 μm thick slices in the cryostat at –20 °C. The air dried samples were fixed with methanol at −20 °C for 10 min and then blocked with horse serum. After the blocking solution was removed, the diluted primary antibody (OX-34) (Santa Cruz) was added to each section and incubated overnight at 4 °C. The next day, the primary antibody was washed away with PBST buffer, and HRP- or Alexa Fluor 555-conjugated secondary antibodies were then used to visualize the presence of protein contrast agents in tumor slides.

### GRPR expression in different tumor tissues by immunohistochemistry staining

The PC3 and H441 tumors from xenografted mice were encapsulated by O.C.T and frozen in liquid nitrogen. The frozen specimens were cut into 5 μm thick slices in the cryostat at –20 °C. PC3 and H441 tumor tissues were stained with primary antibody against GRPR (Cas # ab39883) (abcam) and HRP-conjugated secondary antibody. Substrate solution was added to the slides to reveal the color of the antibody staining.

### Statistical analysis

The statistical analysis of acute toxicity study (supplementary Table 1) was performed using one-tailed *t*-test. No significant statistical differences were detected between control group and mice injected with ProCA1.GRPR. The mouse group sizes were set to support statistically valid data and to minimize the use of the animals. Mice were randomly assigned to groups for the experiments. An experienced pathologist was blinded to the groups of H&E staining (supplementary Fig. 8) to evaluate the organ toxicity of ProCA1.GRPR.

## Additional Information

**How to cite this article**: Pu, F. *et al.* GRPR-targeted Protein Contrast Agents for Molecular Imaging of Receptor Expression in Cancers by MRI. *Sci. Rep.* **5**, 16214; doi: 10.1038/srep16214 (2015).

## Supplementary Material

Supplementary Information

## Figures and Tables

**Table 1:** Gd^3+^ and Zn^2+^ binding affinity and selectivity of Gd-DTPA and ProCA1 variants.

	DTPA	ProCA1	ProCA1.GRPR	ProCA1.B10	ProCA1.G10
K_d Gd_ , M	3.5 × 10^−23^ *	8.7 × 10^−13^ *	2.1 ± 0.6 × 10^−12^	5.1 ± 0.1 × 10^−12^	1.3 ± 0.1 × 10^−12^
K_d Zn_, M	5.1 × 10^−19^ *	1.9 × 10^−7^ *	4.1 ± 2.0 × 10^−6^	9.0 ± 0.1 × 10^−6^	4.6 ± 0.4 × 10^−6^
log (K_Gd_/K_Zn_)	4.2	5.4	6.3	6.3	6.5
r_1_, mM^−1^s^−1^, 1.4 T	3.5	25.9 ± 2.6	42.0 ± 2.6	47.5 ± 0.1	49.2 ± 0.2
r_2_, mM^−1^s^−1^, 1.4 T	5	41.2 ± 3.3	78.9 ± 2.6	80.3 ± 0.7	92.0 ± 0.1

^*^from references^21^.

## References

[b1] Reile H., Armatis P. E. & Schally A. V. Characterization of high-affinity receptors for bombesin/gastrin releasing peptide on the human prostate cancer cell lines PC-3 and DU-145: internalization of receptor bound 125I-(Tyr4) bombesin by tumor cells. Prostate 25, 29–38 (1994). 8022709 10.1002/pros.2990250105

[b2] Reubi J. C., Wenger S., Schmuckli-Maurer J., Schaer J. C. & Gugger M. Bombesin receptor subtypes in human cancers: detection with the universal radioligand (125)I-[D-TYR(6), beta-ALA(11), PHE(13), NLE(14)] bombesin(6-14). Clin Cancer Res 8, 1139–1146 (2002). 11948125

[b3] Gugger M. & Reubi J. C. Gastrin-releasing peptide receptors in non-neoplastic and neoplastic human breast. The American journal of pathology 155, 2067–2076 (1999). 10595936 10.1016/S0002-9440(10)65525-3 PMC1866930

[b4] Markwalder R. & Reubi J. C. Gastrin-releasing peptide receptors in the human prostate: relation to neoplastic transformation. Cancer research 59, 1152–1159 (1999). 10070977

[b5] Jensen R. T., Battey J. F., Spindel E. R. & Benya R. V. International Union of Pharmacology. LXVIII. Mammalian bombesin receptors: nomenclature, distribution, pharmacology, signaling, and functions in normal and disease states. Pharmacological reviews 60, 1–42 (2008). 18055507 10.1124/pr.107.07108 PMC2517428

[b6] Achilefu S. *et al.* Synthesis, *in vitro* receptor binding, and *in vivo* evaluation of fluorescein and carbocyanine peptide-based optical contrast agents. Journal of medicinal chemistry 45, 2003–2015 (2002). 11985468 10.1021/jm010519l

[b7] Cai Q. Y. *et al.* Near-infrared fluorescence imaging of gastrin releasing peptide receptor targeting in prostate cancer lymph node metastases. Prostate 73, 842–854 (2013). 23280511 10.1002/pros.22630

[b8] Ma L. *et al.* *In vitro* and *in vivo* evaluation of Alexa Fluor 680-bombesin[7-14]NH2 peptide conjugate, a high-affinity fluorescent probe with high selectivity for the gastrin-releasing peptide receptor. Molecular imaging 6, 171–180 (2007). 17532883

[b9] Young S. H. & Rozengurt E. Qdot nanocrystal conjugates conjugated to bombesin or ANG II label the cognate G protein-coupled receptor in living cells. American journal of physiology. Cell physiology 290, C728–732 (2006). 16236822 10.1152/ajpcell.00310.2005

[b10] Chang Y. J. *et al.* Molecular imaging and therapeutic efficacy of 188Re-(DXR)-liposome-BBN in AR42J pancreatic tumor-bearing mice. Oncology reports 28, 1736–1742 (2012). 22922965 10.3892/or.2012.1978

[b11] Liu Y. *et al.* A comparative study of radiolabeled bombesin analogs for the PET imaging of prostate cancer. Journal of nuclear medicine : official publication, Society of Nuclear Medicine 54, 2132–2138 (2013). 10.2967/jnumed.113.121533 PMC4215198 24198391

[b12] Kahkonen E. *et al.* *In vivo* imaging of prostate cancer using [68Ga]-labeled bombesin analog BAY86-7548. Clin Cancer Res 19, 5434–5443 (2013). 23935037 10.1158/1078-0432.CCR-12-3490

[b13] Mather S. J. *et al.* GRP receptor imaging of prostate cancer using [(99m)Tc]Demobesin 4: a first-in-man study. Molecular imaging and biology : MIB : the official publication of the Academy of Molecular Imaging 16, 888–895 (2014). 24915934 10.1007/s11307-014-0754-z

[b14] Van de Wiele C. *et al.* Technetium-99m RP527, a GRP analogue for visualisation of GRP receptor-expressing malignancies: a feasibility study. European journal of nuclear medicine 27, 1694–1699 (2000). 11105826 10.1007/s002590000355

[b15] Scopinaro F. *et al.* 99mTc-bombesin detects prostate cancer and invasion of pelvic lymph nodes. European journal of nuclear medicine and molecular imaging 30, 1378–1382 (2003). 12920485 10.1007/s00259-003-1261-7

[b16] Bodei L. *et al.* 177Lu-AMBA Bombesin analogue in hormone refractory prostate cancer patients: a phase I escalation study with single-cycle administrations. European journal of nuclear medicine and molecular imaging 34, S221 (2007).

[b17] Hofmann M. *et al.* Feasibility of Ga-68-DOTABOM PET in prostate carcinoma patients. In European journal of nuclear medicine and molecular imaging, Vol. 31 S253–S253 (SPRINGER 233 SPRING STREET, NEW YORK, NY 10013 USA, 2004).

[b18] Hricak H. MR imaging and MR spectroscopic imaging in the pre-treatment evaluation of prostate cancer. The British journal of radiology 78 Spec No 2, S103–111 (2005). 16306632 10.1259/bjr/11253478

[b19] Winter P. M. *et al.* Molecular imaging of angiogenesis in nascent Vx-2 rabbit tumors using a novel alpha(nu)beta3-targeted nanoparticle and 1.5 tesla magnetic resonance imaging. Cancer research 63, 5838–5843 (2003). 14522907

[b20] Marckmann P. *et al.* Nephrogenic systemic fibrosis: suspected causative role of gadodiamide used for contrast-enhanced magnetic resonance imaging. Journal of the American Society of Nephrology : JASN 17, 2359–2362 (2006). 16885403 10.1681/ASN.2006060601

[b21] Yang J. J. *et al.* Rational design of protein-based MRI contrast agents. Journal of the American Chemical Society 130, 9260–9267 (2008). 18576649 10.1021/ja800736h PMC2692035

[b22] Xue S., Qiao J., Pu F., Cameron M. & Yang J. J. Design of a novel class of protein-based magnetic resonance imaging contrast agents for the molecular imaging of cancer biomarkers. Wiley interdisciplinary reviews. Nanomedicine and nanobiotechnology 5, 163–179 (2013). 23335551 10.1002/wnan.1205 PMC4011496

[b23] Qiao J. *et al.* Molecular imaging of EGFR/HER2 cancer biomarkers by protein MRI contrast agents. Journal of biological inorganic chemistry : JBIC : a publication of the Society of Biological Inorganic Chemistry 19, 259–270 (2014). 24366655 10.1007/s00775-013-1076-3 PMC3931309

[b24] Xue S. *et al.* Design of ProCAs (protein-based Gd(3+) MRI contrast agents) with high dose efficiency and capability for molecular imaging of cancer biomarkers. Medicinal research reviews 34, 1070–1099 (2014). 24615853 10.1002/med.21313

[b25] Ye Y., Lee H. W., Yang W., Shealy S. & Yang J. J. Probing site-specific calmodulin calcium and lanthanide affinity by grafting. Journal of the American Chemical Society 127, 3743–3750 (2005). 15771508 10.1021/ja042786x

[b26] Wei L. *et al.* Protein-based MRI contrast agents for molecular imaging of prostate cancer. Molecular imaging and biology : MIB : the official publication of the Academy of Molecular Imaging 13, 416–423 (2011). 20574851 10.1007/s11307-010-0342-9 PMC3463956

[b27] Zhou Y., Xue S., Chen Y. & Yang J. J. Probing Ca2+-binding capability of viral proteins with the EF-hand motif by grafting approach. Methods in molecular biology 963, 37–53 (2013). 23296603 10.1007/978-1-62703-230-8_3

[b28] Corjay M. H. *et al.* Two distinct bombesin receptor subtypes are expressed and functional in human lung carcinoma cells. The Journal of biological chemistry 266, 18771–18779 (1991). 1655761

[b29] Lantry L. E. *et al.* 177Lu-AMBA: Synthesis and characterization of a selective 177Lu-labeled GRP-R agonist for systemic radiotherapy of prostate cancer. Journal of nuclear medicine : official publication, Society of Nuclear Medicine 47, 1144–1152 (2006). 16818949

[b30] Qiao J. *et al.* HER2 targeted molecular MR imaging using a de novo designed protein contrast agent. PloS one 6, e18103 (2011). 21455310 10.1371/journal.pone.0018103 PMC3063795

[b31] Allison M. The HER2 testing conundrum. Nat Biotechnol 28, 117–119 (2010). 20139941 10.1038/nbt0210-117

[b32] Caravan P. *et al.* Collagen-targeted MRI contrast agent for molecular imaging of fibrosis. Angew Chem Int Ed Engl 46, 8171–8173 (2007). 17893943 10.1002/anie.200700700

[b33] Polasek M. *et al.* Molecular MR Imaging of Liver Fibrosis: A Feasibility Study using Rat and Mouse Models. J Hepatol 57, 549–555 (2012). 22634342 10.1016/j.jhep.2012.04.035 PMC3423553

[b34] Spuentrup E. *et al.* Molecular magnetic resonance imaging of myocardial perfusion with EP-3600, a collagen-specific contrast agent: initial feasibility study in a swine model. Circulation 119, 1768–1775 (2009). 19307474 10.1161/CIRCULATIONAHA.108.826388

[b35] Burtea C. *et al.* Magnetic resonance molecular imaging of vascular cell adhesion molecule-1 expression in inflammatory lesions using a peptide-vectorized paramagnetic imaging probe. Journal of medicinal chemistry 52, 4725–4742 (2009). 19580288 10.1021/jm9002654

[b36] De Leon-Rodriguez L. M. *et al.* MRI detection of VEGFR2 *in vivo* using a low molecular weight peptoid-(Gd)8-dendron for targeting. Journal of the American Chemical Society 132, 12829–12831 (2010). 20795620 10.1021/ja105563a PMC2967214

[b37] Zhu W., Okollie B., Bhujwalla Z. M. & Artemov D. PAMAM dendrimer-based contrast agents for MR imaging of Her-2/neu receptors by a three-step pretargeting approach. Magn Reson Med 59, 679–685 (2008). 18302223 10.1002/mrm.21508 PMC2947957

[b38] Flacke S. *et al.* Novel MRI contrast agent for molecular imaging of fibrin: implications for detecting vulnerable plaques. Circulation 104, 1280–1285 (2001). 11551880 10.1161/hc3601.094303

[b39] Cheng Z., Thorek D. L. & Tsourkas A. Gadolinium-conjugated dendrimer nanoclusters as a tumor-targeted T1 magnetic resonance imaging contrast agent. Angew Chem Int Ed Engl 49, 346–350 (2010). 19967688 10.1002/anie.200905133 PMC2862691

[b40] Ouimet T. *et al.* Molecular and cellular targets of the MRI contrast agent P947 for atherosclerosis imaging. Mol Pharm 9, 850–861 (2012). 22352457 10.1021/mp2003863

[b41] Sun Y. G. & Chen Z. F. A gastrin-releasing peptide receptor mediates the itch sensation in the spinal cord. Nature 448, 700–703 (2007). 17653196 10.1038/nature06029

[b42] Patel O., Shulkes A. & Baldwin G. S. Gastrin-releasing peptide and cancer. Biochimica et biophysica acta 1766, 23–41 (2006). 16490321 10.1016/j.bbcan.2006.01.003

[b43] Asti M. *et al.* Influence of different chelators on the radiochemical properties of a 68-Gallium labelled bombesin analogue. Nuclear medicine and biology 41, 24–35 (2014). 24183610 10.1016/j.nucmedbio.2013.08.010

[b44] Varasteh Z. *et al.* The effect of mini-PEG-based spacer length on binding and pharmacokinetic properties of a 68Ga-labeled NOTA-conjugated antagonistic analog of bombesin. Molecules 19, 10455–10472 (2014). 25036155 10.3390/molecules190710455 PMC6270800

[b45] Pan D. *et al.* PET imaging of prostate tumors with 18F-Al-NOTA-MATBBN. Contrast media & molecular imaging 9, 342–348 (2014). 24729577 10.1002/cmmi.1583

[b46] Varasteh Z. *et al.* *In vitro* and *in vivo* evaluation of a (18)F-labeled high affinity NOTA conjugated bombesin antagonist as a PET ligand for GRPR-targeted tumor imaging. PloS one 8, e81932 (2013). 24312607 10.1371/journal.pone.0081932 PMC3849266

[b47] Jimenez-Mancilla N. *et al.* Multifunctional targeted therapy system based on (99m) Tc/(177) Lu-labeled gold nanoparticles-Tat(49-57)-Lys(3) -bombesin internalized in nuclei of prostate cancer cells. Journal of labelled compounds & radiopharmaceuticals 56, 663–671 (2013). 25196028 10.1002/jlcr.3087

[b48] Zhou Z. *et al.* Synthesis and *in vitro* and *in vivo* evaluation of hypoxia-enhanced 111In-bombesin conjugates for prostate cancer imaging. Journal of nuclear medicine : official publication, Society of Nuclear Medicine 54, 1605–1612 (2013). 10.2967/jnumed.112.117986 PMC4049340 23896558

[b49] Fulton D. A. *et al.* Efficient relaxivity enhancement in dendritic gadolinium complexes: effective motional coupling in medium molecular weight conjugates. Chemical communications, 474–476 (2005). 15654374 10.1039/b413536a

[b50] Fulton D. A. *et al.* Glycoconjugates of gadolinium complexes for MRI applications. Chemical communications, 1064–1066 (2006). 16514440 10.1039/b517997a

[b51] Caravan P., Ellison J. J., McMurry T. J. & Lauffer R. B. Gadolinium(III) Chelates as MRI Contrast Agents: Structure, Dynamics, and Applications. Chemical reviews 99, 2293–2352 (1999). 11749483 10.1021/cr980440x

[b52] Uehara H. *et al.* The molecular basis for high affinity of a universal ligand for human bombesin receptor (BnR) family members. Biochemical pharmacology 84, 936–948 (2012). 22828605 10.1016/j.bcp.2012.07.010 PMC3433740

[b53] Mantey S. A. *et al.* Rational design of a peptide agonist that interacts selectively with the orphan receptor, bombesin receptor subtype 3. The Journal of biological chemistry 276, 9219–9229 (2001). 11112777 10.1074/jbc.M008737200

[b54] Wang L. H. *et al.* Activation of neuromedin B-preferring bombesin receptors on rat glioblastoma C-6 cells increases cellular Ca2+ and phosphoinositides. The Biochemical journal 286 (**Pt 2**), 641–648 (1992). 1326946 10.1042/bj2860641 PMC1132948

[b55] Shrivastava A. *et al.* A high-affinity near-infrared fluorescent probe to target bombesin receptors. Molecular imaging and biology : MIB : the official publication of the Academy of Molecular Imaging 16, 661–669 (2014). 24604209 10.1007/s11307-014-0727-2 PMC5885141

[b56] Tweedle M. F. Peptide-targeted diagnostics and radiotherapeutics. Accounts of chemical research 42, 958–968 (2009). 19552403 10.1021/ar800215p

[b57] Fang J., Nakamura H. & Maeda H. The EPR effect: Unique features of tumor blood vessels for drug delivery, factors involved, and limitations and augmentation of the effect. Advanced drug delivery reviews 63, 136–151 (2011). 20441782 10.1016/j.addr.2010.04.009

[b58] Dreher M. R. *et al.* Tumor vascular permeability, accumulation, and penetration of macromolecular drug carriers. Journal of the National Cancer Institute 98, 335–344 (2006). 16507830 10.1093/jnci/djj070

[b59] Xue S. *et al.* Protein MRI contrast agent with unprecedented metal selectivity and sensitivity for liver cancer imaging. Proceedings of the National Academy of Sciences 112, 6607–6612 (2015). 10.1073/pnas.1423021112 PMC4450423 25971726

[b60] Zhang C. *et al.* Specific targeting of tumor angiogenesis by RGD-conjugated ultrasmall superparamagnetic iron oxide particles using a clinical 1.5-T magnetic resonance scanner. Cancer research 67, 1555–1562 (2007). 17308094 10.1158/0008-5472.CAN-06-1668

[b61] Schmieder A. H. *et al.* Molecular MR imaging of neovascular progression in the Vx2 tumor with alphavbeta3-targeted paramagnetic nanoparticles. Radiology 268, 470–480 (2013). 23771914 10.1148/radiol.13120789 PMC3721054

[b62] Cai W. & Chen X. Multimodality molecular imaging of tumor angiogenesis. Journal of nuclear medicine : official publication, Society of Nuclear Medicine 49 Suppl 2, 113S–128S (2008). 10.2967/jnumed.107.045922 18523069

[b63] Mahmood U., Tung C. H., Bogdanov A. Jr. & Weissleder R. Near-infrared optical imaging of protease activity for tumor detection. Radiology 213, 866–870 (1999). 10580968 10.1148/radiology.213.3.r99dc14866

[b64] Delli Castelli D., Ferrauto G., Cutrin J. C., Terreno E. & Aime S. *In vivo* maps of extracellular pH in murine melanoma by CEST-MRI. Magn Reson Med 71, 326–332 (2014). 23529973 10.1002/mrm.24664

