# Supplementary material for: GRPR-targeted Protein Contrast Agents for Molecular Imaging of Receptor Expression in Cancers by MRI
Source: Sci Rep. 2015 Nov 18;5:16214. doi: 10.1038/srep16214 (PMC4649707; doi:10.1038/srep16214)
Supplement: Supplementary Information [file srep16214-s1.pdf]

Supplementary information

GRPR-targeted Protein Contrast Agents for Molecular Imaging of Receptor Expression in Cancers by MRI

Fan Pu[a], Jingjuan Qiao[a], Shenghui Xue[a], Hua Yang[b], Anvi Patel[a], Lixia Wei[a], Khan Hekmatyar[c], Mani Salarian[a], Hans E. Grossniklaus[b], Zhi-Ren Liu[a] and Jenny J. Yang[a,*]

[a.] Departments of Chemistry and Biology, Center for Diagnostics & Therapeutics, Georgia State University, Atlanta, GA 30303, USA. [b.] Department of Ophthalmology, Emory University, Atlanta, GA, 30322. [c.] Bio-imaging Research Center, University of Georgia, Athens, GA, 30602.

Table of contents:

1. Supplementary materials and methods
2. Supplementary figures
3. Supplementary table
4. Supplementary reference

1. Supplementary materials and methods

Tryptophan fluorescence and circular dichroism spectroscopy of ProCA1 variants.

The conformational analysis of ProCA1 variants was investigated by tryptophan fluorescence spectroscopy and circular dichroism (CD) spectroscopy. The tryptophan emission range is at 300 – 400 nm with its excitation range at 280 nm; free tryptophan is used as a control. The CD spectrum of ProCA1 was obtained by CD spectrums (Jasico, USA). 25 μM of protein in Tris/HCl buffer was loaded into the cell with a 1 mm length. The CD signals were monitored by wavelengths from 190 nm to 260 nm. 25 μM Gd^{3+} was loaded in the cuvette to detect the effects on the protein structure.

Molecular modeling and docking studies.

The model structure of ProCA1.B10, ProCA1.G10 and ProCA1.GRPR were generated by I-TASSER. The model structure of GRPR was also generated by I-TASSER based on Neurokinin 1 receptor (NK1R), a protein in the family B of GPCR with the highest homogeneity with identity of 26% and coverage of 85% to GRPR in the primary sequence. *In silico* docking between GRPR and GRPR-targeted ProCA1 (ProCA1.GRPR, ProCA1.G10 and ProCA1.B10) was performed using HADDOCK based on the interaction pocket for NKIR and the peptide residues Asn3, Val4, Leu5, Asp10, Ile15, Thr17, Thr19, Ser20, and Glu21[1] .

Pharmacokinetic studies.

To further study the pharmacokinetics of ProCA1.GRPR, plasma of mice were collected at different time points post the injection. After the serum was digested with 70% nitric acid, each sample was diluted by 2% nitric acid to 4 ml for ICP-OES analysis. The amount of Gd^{3+} in serum at different time points was determined by comparing the intensity of the test sample to

the intensity on the standard curve generated using the standard gadolinium solution (HIGH-PURITY™ STANDARDS). A set of calibration standards and QC samples (three concentrations in duplicate) were included in each analysis session to generate a standard curve and to assess assay performance. The two-compartment model was used to calculate the half-life ($t_{1/2}$) and volume distribution (V_d) of ProCA1 using KaleidaGraph software.

2. Supplementary figures

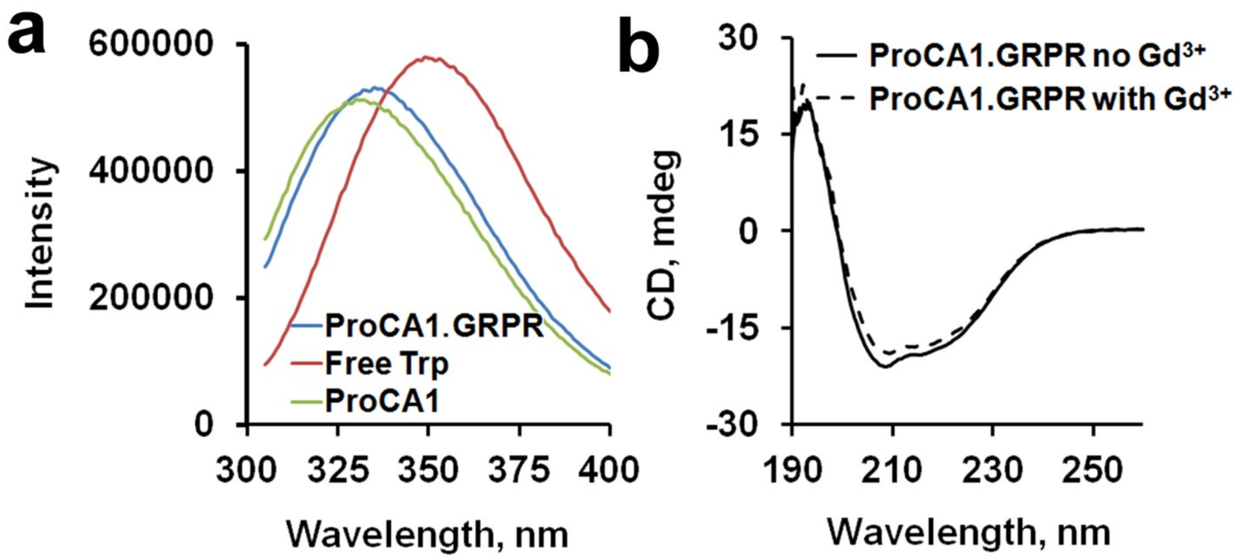

Supplementary Figure 1. Tryptophan fluorescence (a) and circular dichroism spectroscopy (b) of ProCA1 variants.

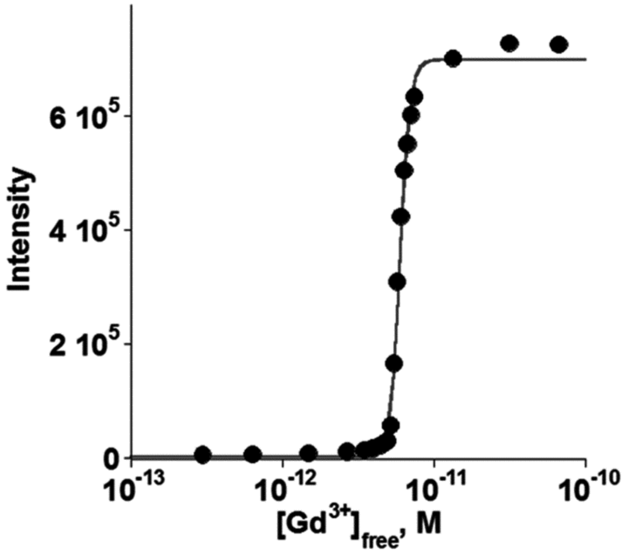

Supplementary Figure 2. Determination of the dissociation constant of Gd^{3+} to Fluo-5N using Gd^{3+}-NTA system.

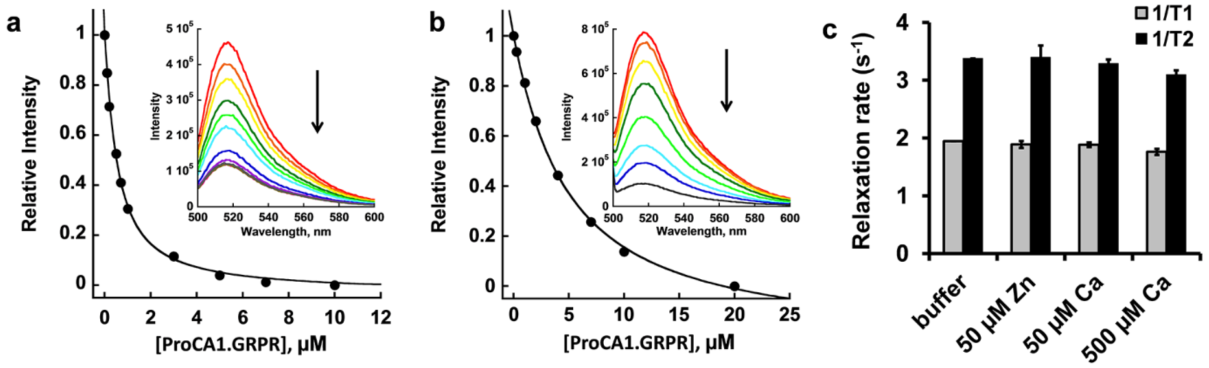

Supplementary Figure 3. ProCA1.GRPR has high Gd^{3+} affinity and selectivity over physiological metal ions, such as Zn^{2+} and Ca^{2+}. (**a**) The determination of the Gd^{3+} binding constant of ProCA1.GRPR was performed by fluorescence spectroscopy. The relative fluorescence intensity of Fluo-5N-Gd complex decreased when we increased the ProCA1.GRPR concentration. The K_d of ProCA1.GRPR to Gd^{3+} was calculated by equation (1). (**b**) The

determination of the Zn^{2+} binding constant of ProCA1.GRPR using fluorescence spectroscopy. The relative fluorescence intensity of FluoZin-1-Zn complex decreased when increasing the ProCA1.GRPR concentration. The K_d of ProCA1.GRPR to Zn^{2+} was calculated by equation (1). (**c**) The relaxation rate of ProCA1.GRPR in HEPES buffer (labeled as buffer) is similar to that in the presence of 50 μM Zn^{2+}, 50 or 500 μM Ca^{2+}. The relaxation rates are measured in 10 mM HEPES, pH 7.0 at 37 °C and 1.4 T.

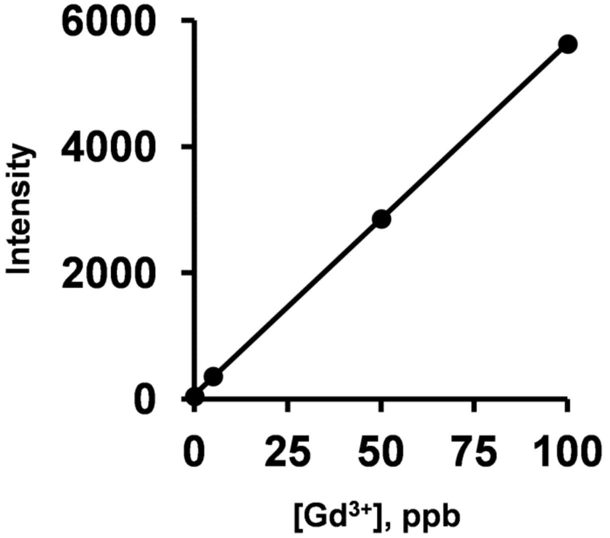

Supplementary Figure 4. The standard curve for ICP-OES to determine Gd^{3+} concentration in mice tissue. The intensity was measured at 342.246 nm at various Gd^{3+} concentrations.

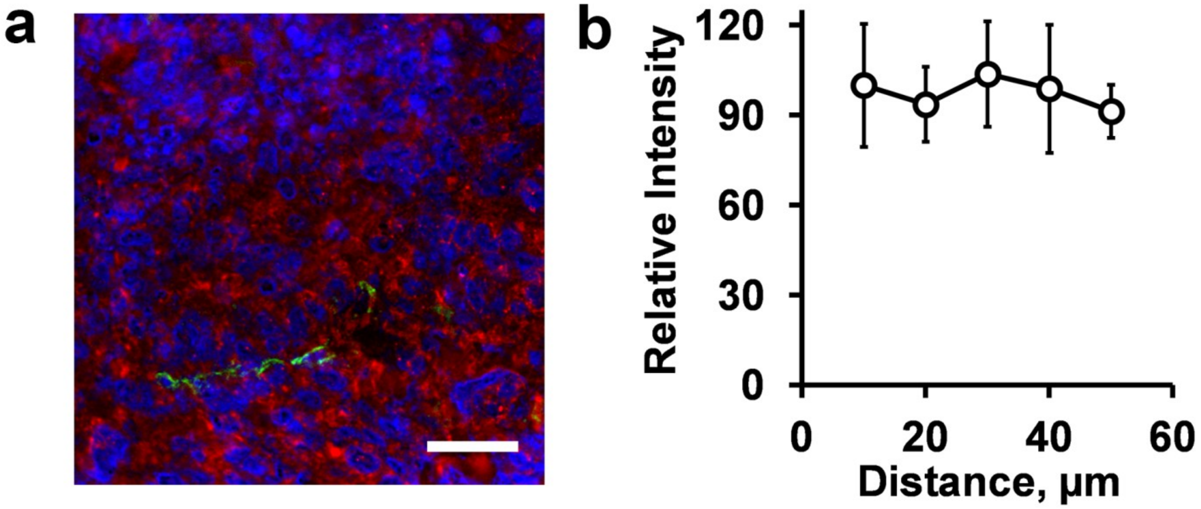

Supplementary Figure 5. ProCA1.GRPR has high tumor tissue penetration. (**a**) Immunostaining of ProCA1.GRPR (red) and CD31 (green) in xenografted PC3 tumor. (**b**) The fluorescence intensity of ProCA1.GRPR is similar at different distances away from blood vessel (CD31 staining) indicating the distribution of ProCA1.GRPR is not restricted in the tumor region near blood vessel. Scale bar = 100 μm.

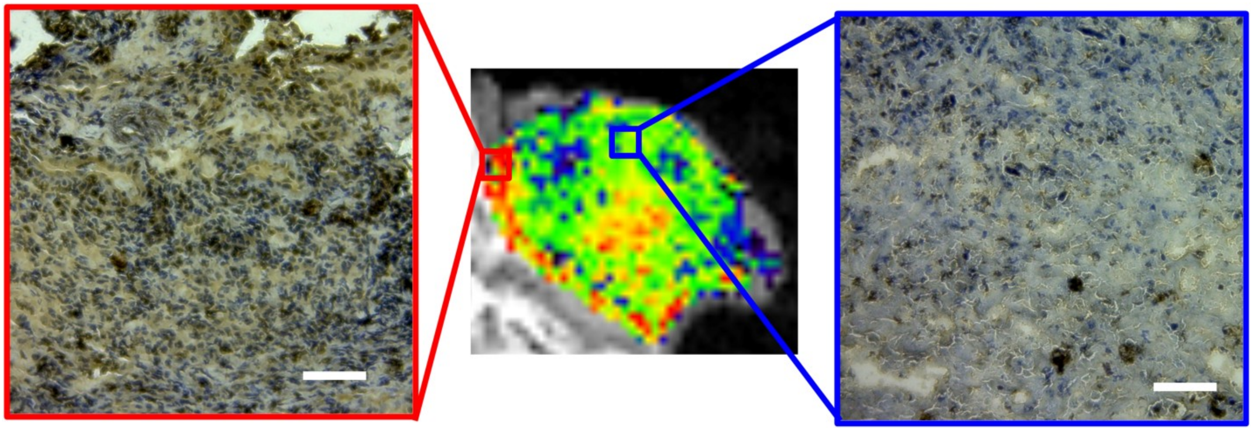

Supplementary Figure 6. Heterogeneous enhancement of PC3 tumor in MRI (mid) correlates well with ProCA1.GRPR distribution using IHC staining. Brown color indicates

the ProCA1.GRPR staining. The tissues with high MRI intensity (red box) shows stronger ProCA1.GRPR staining (red box). The tissue with low MRI intensity (blue box) shows weaker ProCA1.GRPR staining (blue box).

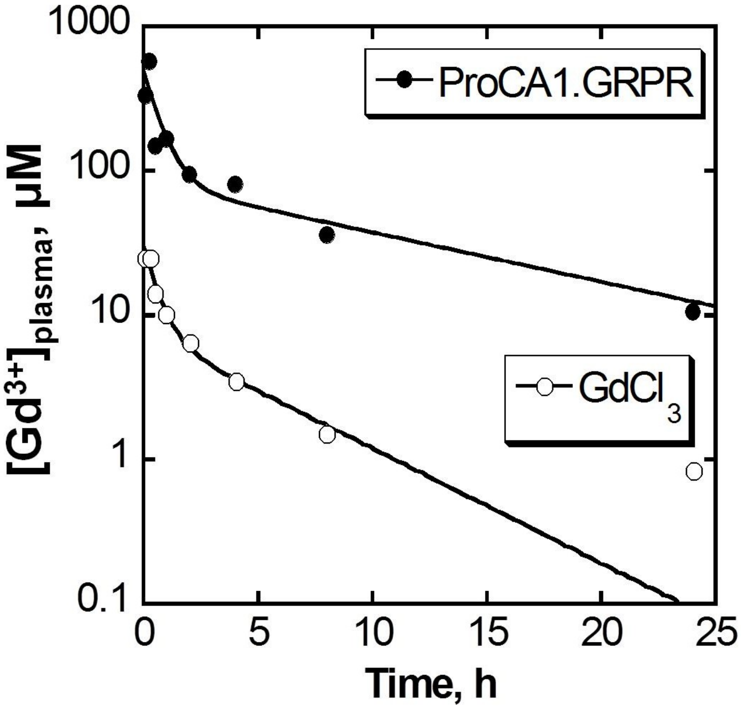

Supplementary Figure 7. Pharmacokinetic studies of PEGylated ProCA1.GRPR and $GdCl_3$. Gd^{3+} concentration in serum was measured by ICP-OES.

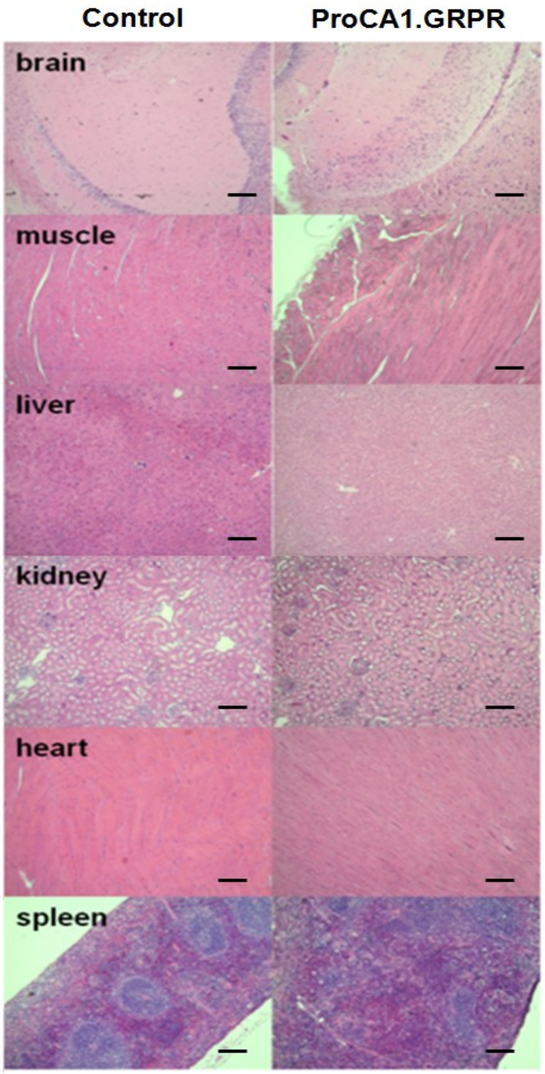

Supplementary Figure 8. H&E staining of mice organs after injection of ProCA1.GRPR or saline (Control) for 2 days. Injection of ProCA1.GRPR did not cause morphological changes in these tissues. Scale bar = 100 µm.

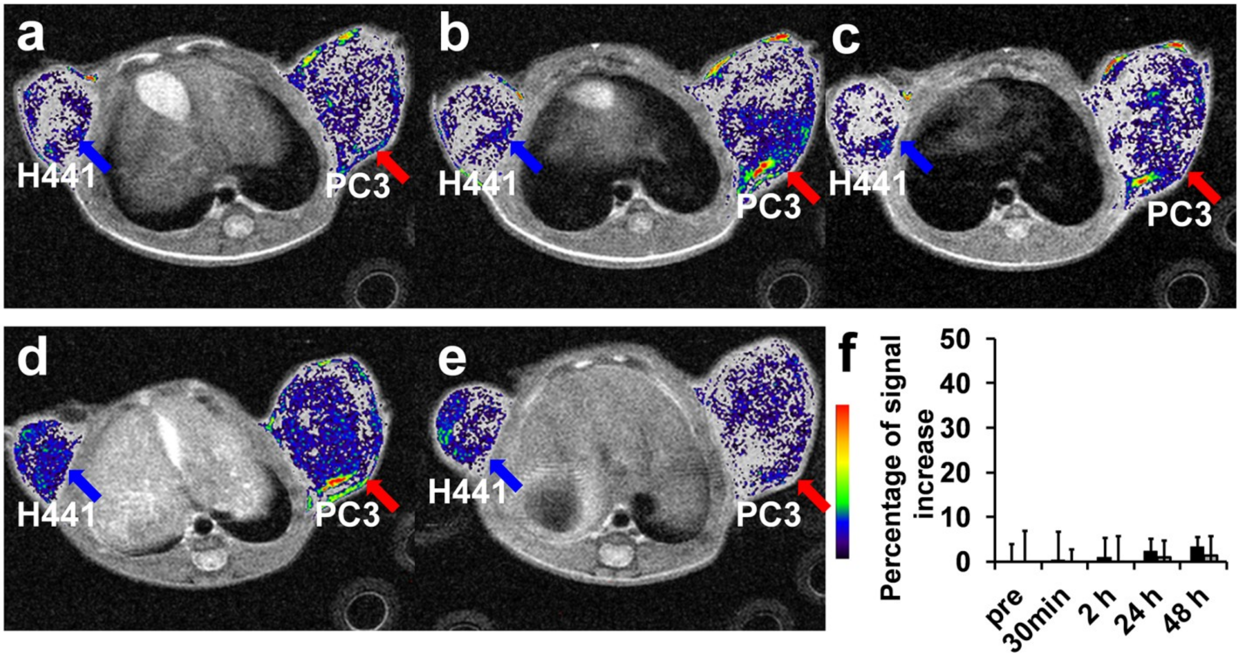

Supplementary Figure 9. T1-weighted MR imaging of xenografted PC3 (red arrow) and H441 (blue arrow) tumors in mice before (a) and after tail vein injection of ProCA1.GRP at 30 min (b), 3 hours (c), 24 hours (d) and 48 hours (e). Tail vein injection of ProCA1.GRP cause less than 5% of signal increase at any time points from 30 min to 48 hours post injection (f).

3. Supplementary table

Supplementary Table 1. The toxicity study of ProCA1.GRPR.

	Blank	ProCA1.GRPR
ALP (U/L)	36.3 ± 4.2	37.0 ± 6.6
AST (U/L)	124.0 ± 9.9	118.5 ± 20.5
Albumin (g/dL)	3.2 ± 0.3	3.1 ± 0.1
Total protein (g/dL)	5.7 ± 0.4	5.7 ± 0.1
Globulin (g/dL)	2.5 ± 0.2	2.6 ± 0.1
BUN (mg/dL)	27.7 ± 5.9	23.7 ± 3.1
Creatinine (mg/dL)	0.1 ± 0.1	0.1 ± 0.1
Cholesterol (mg/dL)	120 ± 21	103 ± 19
Glucose (mg/dL)	257.3 ± 12.4	217.3 ± 24
Calcium (mg/dL)	10.5 ± 0.2	10.3 ± 0.6
Phosphorus (mg/dL)	9.4 ± 1.0	9.1 ± 2.1
Bicarbonate (mmol/L)	27.7 ± 4.2	20.7 ± 5.1
Sodium (mmol/L)	149.3 ± 3.5	151.3 ± 5.0
Chloride (mmol/L)	107.0 ± 0	112.7 ± 2.1

Blood chemistry test of the mice serum collected 2 days post injection of ProCA1.GRPR or saline (blank). The statistical analysis was performed using one-tailed *t*-test. There is no differences in ALP, AST, BUN, creatinine, cholesterol, glucose, Ca^{2+}, Na^{+}, Cl^{-}, PO^{3-}, CO_3^{2-}, albumin, total protein and globubin level between ProCA1.GRPR injected group and control group, indicating ProCA1.GRPR has no toxicity to the mice post injection for 2 days.

4. Supplementary reference

1. Gayen, A., Goswami, S.K. & Mukhopadhyay, C. NMR evidence of GM1-induced conformational change of Substance P using isotropic bicelles. *Biochimica et biophysica acta* **1808**, 127-139 (2011).
